# Sexual Competence in Higher Education: Global Perspective in a Multicentric Project in the Nursing Degree

**DOI:** 10.3390/healthcare9020166

**Published:** 2021-02-04

**Authors:** Daniela Mecugni, Cinzia Gradellini, Ermelinda Caldeira, Vicki Aaberg, Hélia Dias, Sagrario Gómez-Cantarino, Ana Frias, Maria Barros, Luis Sousa, Margarida Sim-Sim

**Affiliations:** 1 EdSex Project, University of Modena and Reggio Emilia, Azienda Unità Sanitaria Locale-IRCCS of Reggio Emilia, 42122 Reggio Emilia, Italy; daniela.mecugni@unimore.it (D.M.); cinzia.gradellini@ausl.re.it (C.G.); 2 EdSex Project, Comprehensive Health Research Centre Integrated Researcher, Nursing Department, University of Evora, 7000-811 Evora, Portugal; ecaldeira@uevora.pt (E.C.); anafrias@uevora.pt (A.F.); mlb@uevora.pt (M.B.); lmms@uevora.pt (L.S.); msimsim@uevora.pt (M.S.-S.); 3 School of Health Sciences, Seattle Pacific University, Seattle, WA 98119, USA; aaberv@spu.edu; 4 EdSex Project, Superior School of Health, Quinta do Mergulhão Srª da Guia, 2005-075 Santarém, Portugal; helia.dias@essaude.ipsantarem.pt; 5 EdSex Project, Campus de Toledo, Nursing, Physiotherapy and Occupational Therapy Department, University of Castilla-La Mancha, 45071 Toledo, Spain

**Keywords:** sexuality, nurse, student, professor, health education, university, care, holistic

## Abstract

Sexuality is an important issue in the university careers of nursing students to ensure that they provide comprehensive care. It is necessary according to the recommendation of the World Health Organization. However, research reveals deficiencies and the need for further development. The aim of the study is to describe the perspective of teachers and students on the content of sexuality in nursing education. The project aims to analyze the attitudes and beliefs of the students about the sexuality of their patients. Furthermore, the experience and sexual lives of the future nurses, as well the teaching of sexuality content in the curriculum, will be analyzed. As for the educators, their level of knowledge about sexuality and vision of sexuality education in undergraduate nursing education will be analyzed. This study is an exploratory and descriptive study with a quantitative-qualitative approach in a multi-center context. The sample is composed of students and professors of nursing courses from five universities (Portugal, Spain, Italy and United States). Questionnaires and semistructured interviews will be used for data collection. The results of the study will allow the inclusion of sexual competence in the curriculum from the beginning in higher education. This article describes the research protocol.

## 1. Introduction

Sexuality is a dimension that characterizes the human being at every moment of life and in every social interaction. Sexuality is best understood not simply as an effect of physiology or the consequence of instinct, but as an expression of existence [1]. Existentialist philosophers, such as Beauvoir, highlighted the restrictions of gender and sexuality, impacting public opinion [2], and Johnson [3] and Kinsey established a comprehensive view of human sexuality and the interaction within and between humans. Kohlberg developed the theory that sexuality begins in childhood; an idea that remains controversial [4]. These scientists and others took sexuality out of the space of intimacy. Sexuality has become a topic of public debate and is considered an expression of existence or a human dimension. In psychology and health sciences, Maslow’s Hierarchy is used, where there are five vital needs, from the most important to the least. In the first place, we find out our physiologic needs, which include sexuality. The importance of its assessment [5] and care is related to the global goal of providing holistic care [6]. When providing care, nurses interact in public, semi-public, or private settings, ideally providing cultural congruent care [7]. Culturally congruent care is particularly sensitive and complex, as the understanding of and expressions of sexuality vary widely. Nurses take care of the human being from birth to death and assist both sexes in the comprehension of different expressions of sexuality and sexual orientation as well as at times of transitional crisis. Knowledge about sexuality is a necessity in those settings, regardless of the geographical location where the care occurs.

Valuing sexuality as a human dimension is not easy as talking about related elements is complex, although nowadays references to sex seem to be everywhere. The complexity of human sexuality makes defining “normal” sexuality problematic. The parameters and standards are difficult or impossible to find because sexual orientation, sexual identity, sexual role, and sexual needs vary greatly from one person to another [8,9].

The World Health Organization (WHO) introduced the concept of sexuality (Education and treatment in human sexuality: the training of health professionals. Geneva, World Health Organization, 1975; WHO Technical Report Series No. 572) for use by health professionals. Sexual wellness is defined by the WHO as the absence of illnesses or problems influencing sexuality and reproduction and the capacity to control and enjoy sexual behaviors, together with freedom from fear, guilt, and harmful beliefs. In addition to this, several factors which influence sexuality must be taken into account: (1) relationship; (2) perception and cognition; (3) values, culture, and beliefs; (4) self-concept; (5) previous experiences; (6) pregnancy; (7) context (e.g., hospitalization, illnesses, or disability), and (8) treatments such as drugs or surgeries [9,10]. Recently, reflecting social movements, the WHO reaffirmed the importance of sexual health, considering sexuality to be an important and integral aspect of human development and maturation throughout the life cycle [10].

These reflections demonstrate that assessment of the sexual dimension of humans requires a high level of competence, as does the management of related counseling and treatment, along with communication of the implications, risks and influencing factors of diseases. 

It is absolutely necessary that nursing students learn to provide holistic care, which includes sexual health care, throughout their education. However, many studies of working nurses have revealed a lack of the appropriate attitudes, knowledge, and skills required to meet this need. The same studies show that university curricula are not designed to provide this critical information [11], despite the strong recommendation of the WHO forty years ago. In one study, more than 80% of teachers considered the students unable to address sexuality topics with patients [10,11], and other studies reveal that nursing students give little attention to sexuality subjects [12,13]. Although sexuality is an integral topic in clinical practice, studies suggest a certain level of discomfort among professionals due to lack of knowledge and discomfort and embarrassment in discussing sexuality [14]. 

Nurses provide care after a process of formal education via each school’s curriculum, beginning with first steps in theory classes and continuing with clinical experiences. Ideally, the professional nurse receives knowledge and gains competence to provide appropriate care of their patients’ sexual health. 

The literature reveals a lack of research regarding the human sexuality content taught in nursing programs. Where this content is lacking it must be developed, as the WHO recommends. Some programs omit the topic of sexuality entirely, while other curricula, focused exclusively on heterosexual orientation, ignore crucial areas of human sexuality (e.g., LGBTQ communities, sexual function, and sexual history). Educators attribute deficiencies in human sexuality education to lack of time and the priority of other content [11]. Studies recommend that nursing education curricula provide information about all sexual orientations as a first step to guarantee equality in health care [14,15].

It is clear that nursing education about sexuality is deficient and requires development. Curriculum guidelines are necessary for the appropriate preparation of nurses [15,16]. Nevertheless, the inclusion of this content in nursing education curricula appears problematic. For the last forty years there has been a wide divergence between what nursing curricula offer and what students really need to know [17]. 

In the European Union, human sexuality must be considered through the lens of family norms, social and cultural patterns, and the educational policies of each country. Sex education standards in Europe require affective and emotional education to be the starting point, in order to foster a holistic approach. This means that sexuality must be presented as a positive element of life and not simply as dangerous and requiring medical interventions. A multidisciplinary approach has recently been suggested for the inclusion of human sexuality issues in schools. Since 2003, sex education has been a compulsory subject in 19 European countries. Three different models of sex education in schools are presented in the literature: (1) education focused on abstinence; (2) abstinence education along with education on safe sex and contraception; (3) education on abstinence, safe sex, and contraception, accompanied by an emphasis on personal growth. Although studies show that the first approach is not effective, it is still used in many countries, including the United States, while the third approach appears to be the most popular in Western Europe [18].

Europe presents a variety of sexuality education practices: in certain countries (e.g., Portugal) human sexuality learning starts at 5 years old and in others (e.g., Cyprus) at 14. In Italy, several legislative proposals have been made to introduce sexuality education, but none has been passed. In Italy, human sexuality education modules are available for students older than 13, but the decision to use these belongs to each school [18,19]. In Spain, there is no legal obligation to provide sexuality content, but sexuality education is carried out through specific programs. However, the Organic Law of Education (LOE) of 2006 promoted equal opportunities between the sexes, the elimination of gender violence and the inclusion of affective-sexual education in schools. However, in 2013, with the Organic Law for the Improvement of Educational Quality (LOMCE), it meant a step backwards, as it eliminated the school subject created by the LOE for affective-sexual education. In Portugal, this instruction is legally required in educational programs. Portugal and Spain are thought to have the best sex education programs, and yet in Spain it seems insufficient for the prevention of STIs. In Portugal, despite good school programs, teenage pregnancy (i.e., births per 1000 women ages 15–19) is still high (i.e., 9.90/1000) and gender equality is not respected, as the number of domestic violence victims reveals [19,20]. In Italy, due to an absence of legal requirement, schools are left to make independent decisions about sexuality education. That is, there may be total omission of sexuality content, or schools may choose to present only the content that they consider of interest. However, most students consider sexuality to be a fundamental topic and desire the inclusion of sexuality topics in their education [21]. In the U.S., two opposing points of view drive approaches to sexuality education. Many view attention to this topic as encouraging promiscuity, while others view human sexuality learning as a critical component of education leading to the wellness of society (i.e., Elizabeth Nash https://www.guttmacher.org/about/staff/elizabeth-nash). 

This study is a very timely contribution to the international study of the state of the teaching of human sexuality in nursing programs. The University of Évora (Portugal), the Polytechnic Institute of Santarém (Portugal), the University of Castilla-la-Mancha (Toledo, Spain), the University of Modena and Reggio Emilia (Italy), and the University of Seattle Pacific (USA) make up the collaborative network.

## 2. Materials and Methods 

This is an exploratory, descriptive study with a quantitative-qualitative approach in a multicentric context that is planned to last two academic years (2020–2021 and 2021–2022). 

The project will be presented to the teaching staff at an official meeting in each of the participating universities. The project will be explained to the students and they will be given the option to participate.

The universities participating in the project have joined because they are involved in including sexual competition in the curriculum. There is also collaboration between these universities and their professors, with postgraduate rotations related to cultural and sexual competence. There have even been doctoral studies related to the social and health context, gender, and sexuality.

A priori, the first inclusion criteria that students must meet in order to participate in the study is to be fully enrolled in the relevant subjects and academic year in which the study begins. As for the teaching staff, they must be linked to their university on a full-time basis. These represent situations that both students and teachers must maintain later during the second year of studies. The main objective is to describe the teaching of human sexuality in nursing programs and to analyze the context of the inclusion or exclusion of this content. 

Specific objectives are as follows.

- To describe the attitudes and the beliefs of students towards patients’ sexuality.
- To describe the attitudes and beliefs of students about sexuality and sexual education.
- To describe students’ perception of their quality of sexual life.
- To analyze students’ perspective on the recognition of human sexuality in the curriculum.
- To describe professors’ knowledge on sexuality.
- To describe the professors’ attitudes towards sexual education at the undergraduate level of Nursing training.

Ethical considerations: the study has been approved by the ethical committee of the University of Évora, with the registration 18175, and by the academic bodies in charge of the other partner universities. The teaching staff and students will be informed of the study verbally, by the researchers (one/two for each university). If they agree to participate, the information will be provided in writing, and it is essential that teachers and students sign the consent before their participation. 

Instruments for data collection: the evaluation instruments are described in the following sections. It is important to note that the universities participating in the project have students enrolled from different areas of their country. Therefore, the socio-cultural characteristics of the sample are representative within the participating universities.

Quantitative data collection from students: The quantitative data from students will be collected with multiple instruments. The entire questionnaire will be self-administered via a paper version. Only the Portuguese partner plans to administer the questionnaire via an online version, by Lime Survey Platform. 

The tool includes the following variables, instruments, and scales:

- Sociodemographic data.
- SABS Scale for Portugal [22], and the original version [23] for the rest of the countries.
- Family Apgar Scale [24].
- Sexuality Attitudes and Beliefs and Sex Education Scale (QACSES) [25].
- Sexual Quality of Life—Male (SQoL-M) [26] and female (SQoL-F) [27].

Permission to use each of the mentioned scales has been requested from the authors.

### 2.1. Sexuality Attitudes and Beliefs Survey (SABS)

Quantitative data will be collected using the Survey of Attitudes and Beliefs about Sexuality (SABS) in English [23] and in its Portuguese version [22]. The questionnaire is a Likert scale with six ratings (from strongly disagree to strongly agree). Possible scores range from 12 to 72 points: higher scores indicate a lower ability to handle the sexual dimension in nursing practice. The scale is one-dimensional, and the original form has a Cronbach’s alpha coefficient of 0.750 and a new test of 0.820 [28]. The Portuguese version has been validated, with the elimination of one item (N° 3) because of its low Cronbach’s alpha value. The Portuguese study exhibited a Cronbach’s alpha coefficient of 0.720 and in the test–retest 0.800 [29]. Overall, the validation process confirms the validity and reliability of the translated instrument [22]. The validation process is underway for the Italian and Spanish versions. 

### 2.2. Family Apgar Scale

The Family Apgar Scale is a tool for the evaluation of the level of function within families [30]. It has been validated for many languages such as Portuguese [24] and Spanish [29]. It is composed of five items, evaluating five domains or parameters: (1) Intra-family Adaptability, (2) Partnership, (3) Growth, (4) Affection, and (5) Resolution, which together correspond to the five letters of the acronym “APGAR”. Each question presents 3 options: almost always (2), some of the time (1), and hardly ever (0). The scores range from 0 to 10 in the categories of: (1) Dysfunctional (e.g., score from 0–3 points), (2) Moderately Dysfunctional (e.g., 4–7 points), and (3) Highly Functional (e.g., 8–10 points) [24,30].

The Cronbach’s alpha was 0.80, in a Brazilian study. The discrimination coefficient was between 0.52 and 0.68, and the criterion validity showed correlation coefficient 0.76 [31]. 

### 2.3. Sexuality Attitudes and Beliefs and Sex Education Scale (QACSES)

The Sexuality Attitudes and Beliefs and Sex Education Scale (QACSES) [25] is a questionnaire composed of 17 items, scored in a 5-point Likert response scale (1 “disagree completely” to 5 “completely agree”). The scale has three dimensions: (1) Gender-Related Beliefs and Contraception (items 4, 5, 6, 7, 11, and 13); (2) Beliefs Associated with Violence in Dating, Gender, and Sexual Behavior (items 1, 2, 3, 8, 9, 12 and 17); and (3) Beliefs associated with a relationship (items 14–16). Items 10, 16, 20, 21, 22, and 23 must be scored inversely. Higher values represent more limiting beliefs and negative attitudes about sexuality and sex education. 

The original, is a Portuguese study [32]. The internal consistency of the instrument’s subscales revealed Cronbach’s alpha indexes ranging from 0.72 to 0.75. The instrument does not exist in other language then Portuguese

### 2.4. Sexual Quality of Life—Female (SQoL-F) 

The Sexual Quality of Life—Female (SQoL-F) [27] includes 18 items, each requiring a 6-point Likert-like response. Respondents choose from 1 (completely agree) to 6 (completely disagree). A higher score means a higher quality of sexual life. The score is obtained from the sum of items after reversing those that are formulated in a positive way (items 1, 5, 9, 13, 16, and 18). 

In the original study, the Cronbach’s alpha coefficient was 0.950 [33]. The Sqol-F has useful in women with spinal cord injuries [34] and Polish women over 60 [35]. As much as possible to see, the SQoL-F is not available in languages like Italian, Spanish, or Portuguese. A cultural adaptation and validation process for those languages is in course. 

### 2.5. Sexual Quality of Life—Male (SQoL-M) 

The Sexual Quality of Life—Male (SQoL-M) [26] is a modified version of the SQoL-F which assesses quality of life in men and contains 11 items. The items, in a 6-point Likert-like response, are scored from 1 (completely agree) to 6 (completely disagree). The score is obtained by the sum, and a higher score indicates a higher quality of life. Results are easily compared with other QoL measures by using the following standardizing formula:

Scale Score = ((Sum of actual items − MIN possible)/(MAX − MIN)) × 100

SQOL-M is a unidimensional instrument, with an excellent internal consistency and a Cronbach’s alpha of.0.82. In the convergent validity SQOL-M, correlated with the Index of Premature Ejaculation domains of satisfaction (0.59) and distress (0.50). Discriminant validity was also good [36]. 

The SQoL-M is available in other languages including Italian and Spanish [26], not in Portuguese.

### 2.6. Qualitative Data Collection from Students

Semistructured face-to-face interviews will be used. We will follow a model of 3 blocks of questions [37]. We will use the interview guide to explore the academic environment. The guide first asks for participation and then personal data: (1) sociodemographic (for example, age, social status, and current family) and (2) academic environment (for example, year of course, favorite areas). Other questions obtain information about the teaching of human sexuality, including the characteristics of the courses and their quality. The fifth and final question asks about specific experiences as students in the classroom and clinical settings. Approximately six students from each university represented in the network (Portuguese, Spanish, Italian, and American), will complete the interview, giving a total of 24 participants.

### 2.7. Quantitative Data Collection from Teachers

Teachers will complete a questionnaire that includes a demographic data section (requesting five different items of sociodemographic and professional data) and 16 questions on the topic (Knowledge of sexuality) [38]. The Sexual Knowledge survey has a maximum score of 100 points. The questionnaire includes a survey score key. Each participant will complete the questionnaire using their personal computer or on paper. The estimated time required to complete the questionnaire is 10 min. The authors have been asked for permission to use each of the cited scales.

### 2.8. Qualitative Data Collection from Teachers

We will explore the professional environment of teachers through face-to-face semistructured interviews using an established interview model [37,38]. Participation is first requested and then the following data is collected: (1) sociodemographic (e.g., age, social status, and current family) and (2) academic (e.g., training and teaching experience). Other questions address the teacher’s readiness to teach human sexuality topics, the curricular teaching of the topic, personal effort in teaching the topic, and the teachers’ perception of student comments. The fifth and final question addresses the teacher’s experience in teaching content on human sexuality in the clinical setting [38]. Five professors from each university represented in the group will complete the interview, for a total of 20 participants.

### 2.9. Estimated Sample Size

Three kinds of sample size estimative will be considered: (a) sample size estimative for the quantitative study applied to the students, (b) sample size estimative to the qualitative study applied to students, and (c) sample size estimative to the qualitative study applied to faculty.

### 2.10. Sample Size Estimation for Students’ Quantitative Study

**Figure 1:**
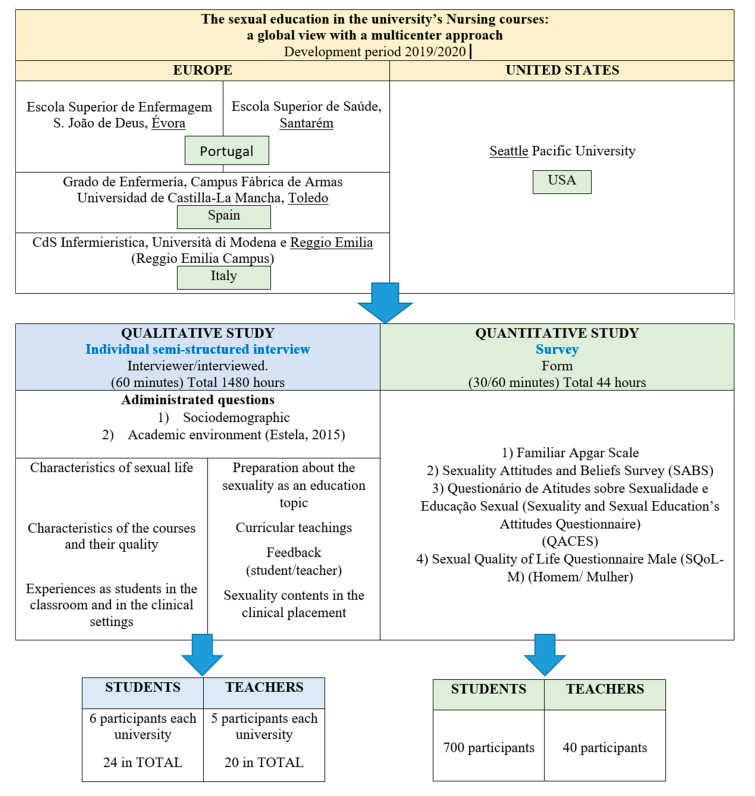
Project plan. Source created by the authors.

Sample size estimative is performed considering the number of students who annually attend the schools. The sum of the number of students attending schools (Ni: N1, N2, N3, N4, N5) is identified as N, the population. Applying the criteria of Almeida et al., [39], the total sample (n) will be about 700 students (Figure 1). The sample in each school (ni) will be performed as a stratified sample, with proportional distribution troughs the formula:

ni = n × (Ni/N). By courses, the same rule of will be applied.(1)

### 2.11. Estimation of the Sample Size for the Qualitative Study of Students

The estimated qualitative study sample for students will be an intentional sample. The sample is organized by focus groups, with 4–5 students [39]. Each school will have a focus group, reaching about 24 students.

### 2.12. Sample Size Estimate for the Qualitative Study of Teachers

The sample of qualitative study will perform as a purposive sample, considering the interest to obtain rich cases. Once the aim is to provide illumination of the phenomena under investigation, sample is not exactly predetermined. Data saturation will show the need to enlarge sample size, or not [40]. Considering Marshall orientation (1996), it will be a convenience sample, developed by stages. The first stage will identify key informants. It will be recruited 20 professors: a sample of 5 Portuguese, 5 Spanish, 5 Italian, and 5 from USA will be invited to participate. These professors will be identified by the profile of teaching students, about the theme (whom teach nursing subjects, where sexuality is relevant, as it can be understandable by syllabus in each curricular unit). The second stage will perform in-depth interviews to the 20 professors, to achieve an interpretative framework of the phenomena, once themes will emerge. Supported by the framework, data continue to be collected until achieve data saturation, which is ordinarily achieved by 15–20 interviews. The third stage will bring a focus group (up to 5 professors per group) to collect data produced by personal interaction.

### 2.13. Sample Selection to the Qualitative and Quantitative Study Applied to Students

Three types of sample selection will be considered: (a) sample selection for the quantitative study applied to students, (b) selection of the sample for the qualitative study applied to the students, and (c) selection of the sample for the qualitative study applied to the teaching staff. We will use a stratified sample for convenience, with a proportional distribution. The research topic, objectives, and data collection methods will be presented to students, personally in class. The group of student volunteers will sign the informed consent that is the first page of the questionnaire. Inclusion criteria for students: age between 18 and 24 years and be a native speaker of the language of each school where data is collected. The exclusion criteria will be: students with children and duration of studies at the nursing school beyond 6 years.

### 2.14. Sample Selection to the Qualitative Study Applied to Students

We will use a convenience sample. By inviting students, we will achieve a group of students that is voluntarily available to share their ideas. The research topic, objectives, and data collection methods will be presented to students in person. A separate text, informed consent, will be entered in the first data collection episode.

The inclusion criteria for students: age between 18 and 24 years old and be a native speaker of the language of each school where the data is collected. The exclusion criteria will be: students with children, and length of study in nursing school beyond 6 years. 

### 2.15. Sample Selection Criteria for Professors’ from Qualitative Study

The professors’ sample will represent approximately 25% of the number of professors teaching in the schools. The research subject, objectives and data collection methods will be presented individually, in the first contact and invitation to professors. The informed consent will be introduced in the first data collection session. 

The inclusion criteria will be (a) at least 2 years of experience as a nursing teacher due to the students’ teaching training and (b) native command of the language of each university where the data will be collected. The exclusion criterion will be to teach less than three hours a week in the classroom, as the average working hours is ten.

### 2.16. Data Collection and Analysis of Data

Depending on the study time, data collection and analysis will be developed. The next section explains the process.

### 2.17. Data Collection and Analysis of Students from Quantitative Data

Data collection will be done using the pencil and paper method. After recruitment for a classroom, the sample will complete the questionnaires. After completing an answer, each student will introduce their own questionnaire, in a box, which will be open, in a private room, to collect data. Depending on the normality or non-normality of the data distribution, parametric or nonparametric tests will be applied. The treatment and analysis of the data will be carried out in IBM^®^ (New York, NY, USA) SPSS^®^ Software, version 24. Software used in the current study has a formal license, granted annually by the University of Évora, to teachers and students The data will be entered in IBM^®^ SPSS^®^ Software by the teacher, who in each school is responsible for the project.

All questionnaire data will be entered into IBM^®^ (New York, NY, USA), SPSS^®^ Software, except those that manifestly show incorrect completion. Software used in the current study has a formal license, granted annually by the University of Évora, to teachers and students. Questionnaires that are fully filled out will be considered for the study.

### 2.18. Data Collection and Analysis of Students from Qualitative Data

Data collection will be done by audio tape, in a private room. The meeting will be defined with the agreement of all the participants. Once the session is over, the audio tape will be transcribed, considering all the speech of all the participants. The data will be entered, in the NVivo software, by the teacher, who in each school is responsible for the project. Data analysis will be done by map coding, dendrograms, and Jaccard analysis.

### 2.19. Collection and Analysis of Teacher Data from Qualitative Data

One teacher at a time will bring the interview. The data will be collected by audio tape, in a private room. The meeting will be defined with the agreement of the teachers. Once the session is over, the audio tape will be transcribed, considering all the speech of all the participants. The data will be entered, in the NVivo software, by the teacher, who in each school is responsible for the project. Data analysis will be done by map coding, dendrograms, and Jaccard analysis.

### 2.20. Confidentiality Will Be Maintained

Confidentiality will be ensured. Instruments are filled automatically and recorded. No name or other variables will identify the participants. If they ask additional questions about the research objectives, they will be given. The results of the study will be published in the nursing literature at the end of the study, but the identities of the participants will not be revealed.

Questionnaires will be destroyed after submission and acceptance by a research journal. However, questionnaires rejected due to incorrect completion, as well as audio tape, will be kept for seven years.

## 3. Discussion/Conclusions

The results of this study will further the development of the teaching of sexual health in the nursing curricula from the beginning of undergraduate nursing study, which will make possible a high level of knowledge and skills for the provision evidence-based health care. Therefore, as indicated by Aaberg [11], nurse educators will be able to teach sexuality content comprehensively throughout the course of undergraduate nursing study. 

Nurse educators must stop acting as transmitters of knowledge and promote student-centered and culturally congruent learning [7]. In addition, educators should adopt and implement a professional training model [14] that includes developing the ability to engage sexual content with patients [41,42]. These active learning activities will reinforce knowledge and build confidence [15]. Nursing professionals have tools to assess people’s sexuality and reproductive development. These instruments have solid theoretical bases such as the care models of Faye Glenn Abdellah, Jean Watson, Virginia Henderson, or Hildegard Peplau [43]. Even nursing has tools to assess the sexuality of patients, such as Gordon’s Functional Patterns or the Nursing Diagnoses of the American Society for Nursing Sciences. These allow nursing professionals to analyze the sexual dimension of the patient in greater depth and relevance [44,45]. Although nursing attaches great importance to sexuality, human sexuality is not yet perceived as an area of its own [46].

This international multi-setting study is critical, because it will provide scientifically verified accounts of the integration of sex education into nursing education for those directly involved. This study will assess the integration of sexuality education within nursing education from the perspective of both the teachers and students in multiple academic settings. It will contribute to the development of appropriate sexuality education, a fundamental part of preparing nursing graduates to provide holistic care. Nursing professionals must have the necessary resources to disseminate scientific knowledge about sexuality to the target population [46,47]. Through an early and comprehensive health intervention focused on health education and the active participation of the population, sexuality education is promoted [48,49]. Thus, nursing principles in the exercise of care are a prerequisite for the improvement of human sexuality.

### Support

This protocol has been prepared with the financial support of a postdoctoral fellowship from the Tordesillas University Group, with the aim of strengthening cooperation in scientific research and promoting structures and networks that promote research in nursing care. (EdSex Research Group).
